# Electronic Cigarette Harms: Aggregate Evidence Shows Damage to Biological Systems

**DOI:** 10.3390/ijerph20196808

**Published:** 2023-09-22

**Authors:** Stephen L. Hamann, Nipapun Kungskulniti, Naowarut Charoenca, Vijj Kasemsup, Suwanna Ruangkanchanasetr, Passara Jongkhajornpong

**Affiliations:** 1 Tobacco Control Research and Knowledge Management Center, Bangkok 10400, Thailand; slhamann@gmail.com (S.L.H.); vijj.kas@mahidol.ac.th (V.K.); suwanna.rua@mahidol.ac.th (S.R.); passara.jon@mahidol.ac.th (P.J.); 2 Faculty of Public Health, Mahidol University, Bangkok 10400, Thailand; nao.naowarut@yahoo.com; 3 Thailand Health Promotion Institute, Bangkok 10330, Thailand; 4 Department of Ophthalmology, Faculty of Medicine, Ramathibodi Hospital, Mahidol University, Bangkok 10400, Thailand

**Keywords:** unsafe, electronic cigarettes, e-cigarettes, health effects

## Abstract

Evidence of the harms of e-cigarettes has been unfolding slowly and has been documented in many reviews and reports worldwide. A narrative review of new evidence is presented since, as research has continued, newly aggregated evidence of the dangers of electronic cigarettes on the brain, heart, and lungs is vital to inform decisions on restricting the use of e-cigarettes. Several biomedical research databases were searched for electronic cigarette health effects, emphasizing reviews, systematic reviews, and meta-analyses. Over 50 review studies, primarily in 2022 and 2023, illustrate some of the latest information on e-cigarette harms. Results show studies of respiratory, neurological, and cardiovascular effects. Researchers call for expanding studies through new methods to elaborate on initial findings of multiple harms emerging in clinical investigations. Since the use of electronic cigarettes for adult cessation is not sanctioned in most countries, it is clear that health authorities see significant costs to the health of the general population if the promotion and use of electronic cigarettes occur worldwide. Regulatory action to control electronic cigarettes should consider the substantial evidence of electronic cigarette harm.

## 1. Background

In 2020, the Tobacco Control Research and Knowledge Management Center (TRC) published “Hidden Dangers of E-Cigarettes”, a book reviewing 75 research reports on the harms of e-cigarettes through 2018 [1]. Much past research was in vitro or in vivo studies or centered on smoking cessation.

In 2022, an extensive review by Australian researchers was published, which included evidence from six independent reviews on the health effects of e-cigarettes from 2018 to 2021 [2]. This review incorporated information from “the 2018 United States (US) National Academies of Sciences, Engineering and Medicine (NASEM) review; the 2018 Public Health England review with an evidence update in 2020; the literature review by the Commonwealth Scientific and Industrial Research Organization (CSIRO) of Australia; the 2020 Irish Health Research Board literature map; the European Union Scientific Committee on Health, Environmental and Emerging Risks (SCHEER) 2021 Opinion on electronic cigarettes; and the US Preventive Services Task Force (USPSTF) 2021 recommendations and evidence synthesis on interventions for tobacco cessation. This Australian systematic review of the National Center for Epidemiology and Population Health for the Australian Department of Health”. It is over 300 pages with 821 references and is a valuable reference for public health concerns about e-cigarette use. Its evidence synthesis of 189 studies has a scope and inclusion period more expansive than we present here.

The aim of this review, which uses the terms electronic cigarettes or e-cigarettes interchangeably, is not to restate evidence in the extensive Australian review but to focus on research evidence from even more recent investigations and reviews up to early 2023, where new evidence of the dangers of e-cigarettes has been provided. This narrative review highlights adverse effects on the respiratory, cardiovascular, and neurological systems and actions necessary to prevent e-cigarette harm.

Worldwide evidence of e-cigarette use shows a 46.6% increase in e-cigarette use in the United States from 2020 to 2022. Based on this rapid increase, the Centers for Disease Control (CDC) called for “comprehensive restrictions on the sale of all flavored tobacco products that include e-cigarettes, menthol cigarettes, and flavored cigars in all jurisdictions.” They emphasized that this strategy, coupled with longstanding evidence-based strategies to prevent youth tobacco use, is expected to reduce youth initiation and use and disparities in tobacco product use [3]. In Thailand, where there is a ban on e-cigarette imports and sales, and in countries with restrictions on the age of sale, advertising, product flavoring, or other sales provisions, there is lower use [4,5].

## 2. Methods

We conducted an overview of the latest evidence in the literature using Pub Med and Google Scholar searches on the health effects of e-cigarettes on the respiratory, cardiovascular, and neurological systems since research on biological system damage from e-cigarettes is expanding. We excluded research focusing on cessation, organizational and regulatory policy, and perceptions and acceptance of electronic cigarettes. However, some studies touch on these topics in reporting health effects on biological systems. Since we were interested in the newest findings, we excluded most clinical research with limited study subjects and studies before 2021. We searched terms such as electronic cigarettes or e-cigarettes, respiratory, cardiovascular, and neurological systems, and lung, heart, and brain effects. The initial results were extensive but were reduced to a limited number when we searched for reviews and systematic reviews with our exclusions. We were interested in systematic reviews since these reviews aggregate relevant studies and have more robust analyses focusing on the pathophysiology of biological systems. Our reported findings are purposive since we focus on harms from electronic cigarettes, and some investigations do not report harms or focus on harms where findings are equivocal or repeat unremarkable characteristics of tobacco or nicotine products. We report over fifty recent review studies with information on their significance in Table 1, Table 2 and Table 3.

## 3. Evidence of Adverse Effects from E-Cigarettes

Several 2021 review studies have documented the detrimental effects of electronic cigarettes [6,7]. These reviews see constant updates as evidence keeps building, showing e-cigarette harms [8]. It is helpful to see some evidence detailing the research on vital organ systems showing evidence that e-cigarettes are unsafe.

### 3.1. Respiratory Effects

See Table 1 for a list of materials on respiratory harms in the manuscript.

Respiratory studies have extended the understanding of e-cigarette components harmful to the lungs. “Toxic substances detected in e-cigarettes include toxicants (chemicals, nanoparticles, and heavy metals) and toxins (endotoxins and beta-glucans). Overall effects include increased cytokines and chemokines, infiltration and activity of inflammatory cells, increased reactive oxygen species (ROS) and DNA damage, and altered cellular mechanisms. Lung inflammation and injury are complex, so multiple factors such as frequency of vaping, e-device type, e-liquid composition, age, sex, and underlying health conditions may affect health outcomes [9].” Research has confirmed exacerbations of asthma, bronchitis, and respiratory tract irritation [10,11,12]. Vaping complex mixtures of certain chemicals and flavorings has been shown to cause adverse acute and long-term effects. Adding substances such as tetrahydrocannabinol (THC) and other potent drugs also complicates results that may arise due to the unregulated nature of vaping in many countries [13,14].

Recent research reviews have highlighted the need for multiple perspectives in assessing findings pointing to risks from e-cigarettes. Hence, there is clarity in comparing and contrasting these risks to those from conventional cigarettes and other nicotine products [6,15,16].

Several recent reviews summarize the adverse consequences of e-cigarette use by illustrating the pathophysiology of vaping lung injury, where multiple mechanisms of injury are involved [9,17,18]. Among the evidence documenting damage to the respiratory system from electronic cigarettes, several investigations have highlighted the unique damage from electronic cigarettes [19,20]. These research examples highlight gene dysregulation and inflammation, which are essential in disease development, and the unique role of e-cigarettes in the development of constrictive bronchiolitis. Since different generations of electronic cigarettes are currently available, research has found distinctive airway damage from different types of e-cigarettes, with the need to continue research as different types of vaping products and ingredients are provided legally and illegally worldwide [21]. Adherence to research guidelines with the specification of controls to eliminate former smokers and the types and duration of e-cigarette use has also been of concern for the quality of research findings [22]. A 2023 scoping study of biomarkers states that “ENDS and e-liquid characteristics as well as use patterns may be associated with elevated exposure to volatile organic compounds (VOCs) and metals. Additional rigorous, controlled studies can assess biomarker exposures from ENDS use and inform the overall risk–benefit of ENDS use for different user populations” [23].

Another scoping review highlights dual use affecting respiratory health and suggests that more significant attention be given to nicotine products’ different harmful constituents [24]. For example, e-cigarettes produce aldehydes, such as acetaldehyde, acrolein, and formaldehyde, and acrolein might “mediate E-cigarette vapour-induced oxidative stress and cell death”, which has also gotten the attention of researchers [25].

Because of dual use, the use of traditional and e-cigarettes is so common, and research in an animal model was performed to determine if substituting e-cigarettes in part might attenuate acute lung injury. However, it did not find any attenuating effect. [26]. Pisinger and Rasmussen reviewed 49 studies of dual users (those using conventional and e-cigarettes). They found that most prospective and cross-sectional studies showed dual use to be at least as harmful as exclusive smoking of traditional cigarettes [27]. A longitudinal study of transition outcomes among adult dual users in the US found “that in a real-world scenario, cigarette and e-cigarette use may hinder rather than facilitate smoking cessation among those interested in quitting [28].”

Because electronic cigarette use has increased lung damage in young people, respiratory specialists have given much attention to the various pro-inflammatory effects of e-cigarettes. Electronic cigarettes and dual use by youth have been studied in 32 European countries to assess approaches to limit use [29]. Findings from a study of maternal e-cigarette use suggest that maternal use may be harmful to offspring development [30]. Pediatric studies have found that e-cigarette use by youth has negative physical and mental health consequences, and there has been an effort to restrict e-cigarette use by youth because of findings of damage to the upper aerodigestive tract [31,32]. For example, the US FDA has been urging pediatricians to perform screenings of youth for e-cigarette product use with preventive messages and appropriate treatment options when use is discovered [33].

Using Positron Emission Tomography (PET), Wetherill and colleagues found that “e-cigarettes produced more significant pulmonary inflammation than conventional cigarettes or controls, with a positive association between pulmonary and peripheral measures of inflammation [34].”

Although extensive examination of electronic cigarettes’ toxicity mechanisms and signal pathways has been completed, there is a need to standardize the frameworks and tools to evaluate e-cigarette impacts on lung health [35]. Zhang and Chandra have suggested ways and tools to evaluate e-cigarettes, and these may be helpful in further assessments [36,37].

### 3.2. Cardiovascular Effects

See Table 2 for a list of materials on cardiovascular harms in the manuscript.

Multiple reports from 2019 to 2022 have documented the accumulating findings of harm to the cardiovascular system. For example, extensive cardiovascular effects include findings of increased arterial stiffness, blood pressure, oxidative stress, myocardial fibrosis, and coronary vascular disease. E-cigarette use also lowers myocardial blood flow on demand, endothelial function, and nitric oxide production [38,39,40,41,42,43].

“Cardiovascular studies reported sympathetic activation, vascular stiffening, and endothelial dysfunction, which are associated with adverse cardiovascular events…. Associations were reported between e-cigarette use and higher incidence of asthma (OR range = 1.39–3.41 [95% CI range: 1.15–6.49]), respiratory disease (OR range = 1.31–2.58 [95% CI range: 1.03–4.89]), COVID-19 (OR = 5.05 [95% CI: 1.82–13.96]), wheeze (OR = 1.67 [95% CI: 1.23–2.15]), and myocardial infarction (OR = 1.79 [95% CI: 1.20–2.66]). Furthermore, dual use of e-cigarettes and cigarettes has been associated with higher rates of cardiovascular disease (OR = 1.36 [95% CI: 1.18–1.56]) and cardiovascular risk factors, including metabolic syndrome (OR = 1.57 [95% CI: 1.03–2.40]) versus sole cigarette users” [39,40].

Cardiac-related effects from e-cigarette use include “multiple acute hemodynamic changes, including higher arterial stiffness, impaired endothelial function, and increased blood pressure, heart rate, and sympathetic tone. Long-term e-cigarette use is also associated with increased arterial stiffness and sympathetic tone” [44]. “Cardiac effects also include reduced myocardial blood flow augmentation with exercise but not changes in ventricular relaxation” [42]. “In addition, short-term e-cigarette use raises the levels of biomarkers of oxidative stress” [43]. “Many acute vascular effects appear to be attributable to nicotine exposure, which is the one chemical tobacco companies have not been interested in removing from their products” [44].

Tobacco interests have sought to boost nicotine in their products by adding ammonia and other ingredients. With e-cigarettes, greater nicotine exposure was generated using nicotine salts that can release a higher nicotine concentration than free-base products. Research has suggested this is why mod-based products have higher nicotine exposure, including disposable e-cigarettes [40].

There is also evidence that flavorings in e-cigarettes can result in cardiovascular damage. Given the multiple effects of electronic cigarettes, the review by Moshensky recommends actions “to reduce youth access …, including removing all flavored e-cigarettes from the market. He suggests that the marketing of e-cigarette products to youth be curtailed to the extent allowable under the law, and young people must be better educated about the harmful effects of e-cigarettes and tobacco products” [45].

A review by Meng and colleagues of eight studies found that “acute inhalation of e-cigarettes leads to negative changes in vascular endothelial function.” Authors conclude that “e-cigarettes cannot be used as an alternative to public health strategies for tobacco control and should not be considered cardiovascular safety products” [46].

Qiu et al. and Carll et al. found that e-cigarette aerosols can “induce arrhythmogenic substrates involved in cardiac electrical, structural, and neural remodeling, facilitating the development of arrhythmias.” They note that findings “provide fresh evidence that the use of e-cigarettes could interfere with normal heart rhythms” [47,48].

Two studies in *Arteriosclerosis, Thrombosis, and Vascular Biology*, one by Mohammadi and colleagues and the other by Nabavizadeh and colleagues, reported endothelial dysfunction through blood changes and vagus nerve signaling, respectively, providing an increased risk of future cardiovascular events in otherwise healthy people [49,50].

A study by Majid and colleagues showed that “Pod-based Electronic Cigarette (PEC) use had comparable short-term and long-lasting impacts on vascular function, BP, and HR as conventional cigarette use among young and healthy adults. Findings indicated that PECs release volatile organic compound (VOC) chemicals that have toxic effects on the blood vasculature” [51].

One recent study shows how immunological changes can affect the risk of cardiovascular disease. Another study highlights how using e-cigarettes affects blood flow in the middle cerebral artery [52,53].

Finally, Mears and colleagues review the multiple effects of e-cigarettes as part of an ongoing review of evidence. They note that “…data is beginning to show that e-cigarettes can cause both short- and long-term issues on cardiac function, vascular integrity, and cardiometabolic issues” [54].

This assessment is upheld in a systematic review by Rahman and colleagues, who note how the risk of severe cardiac conditions increases with e-cigarette use [55]. Siddiqi and others find cardiovascular hemodynamic measures and biomarkers associated with e-cigarette use [56]. Alarabi and colleagues highlight that toxic constituents in e-cigarettes are known to affect inflammation, oxidative stress, platelets, coagulation, and the vascular endothelium, which contribute to critical processes in developing thrombosis [57].

A review of toxicological studies points to adverse health effects but notes that toxicological assessments must keep pace with factors such as e-liquid composition, physical device factors, the health of e-cigarette users, and the chemical changes from different levels of heat produced by e-cigarettes [58].

### 3.3. Neurological Effects

See Table 3 for a list of materials on neurological harms in the manuscript.

Extensive neurological investigation has shown “that nicotine’s reinforcing properties are primarily mediated by the activation of the brain’s mesolimbic reward circuitry and the release of the neurotransmitter dopamine that contributes to the development of addiction. Beyond the brain, nicotinic acetylcholine receptors (nAChRs) are also highly expressed in peripheral neurons, epithelia, and immune cells, where their activation may cause harmful effects” [59,60]. Recent research shows that mental health problems, like depression and schizophrenia, are associated with nicotine use. Susceptibility to nicotine addiction has also been shown to affect brain circuits involved in adverse Attention Deficit/Hyperactivity Disorder (ADHD) outcomes [61,62]. Also, research on how nicotine affects brain development and circuitry may provide new approaches to cessation through brain stimulation. In the meantime, more aggressive measures are being proposed to limit e-cigarette use and nicotine levels in cigarettes to reduce addiction and improve brain function [63].

Not only is the use of e-cigarettes damaging to the respiratory, cardiovascular, and neurological systems, but it also results in addiction to nicotine, a drug associated with long-term multiple-drug use. Nicotine uptake by the brain from e-cigarette use is rapid and can maintain addiction, similar to combustible cigarettes [64]. Studies by Dai and Blagev show that longitudinal nicotine exposure with or without advanced disease consequences has adverse neurocognitive consequences [65,66]. Many studies have confirmed a “gateway effect” from early nicotine use, and many pod-based nicotine products, like Juul and disposable e-cigarettes, have high levels of nicotine that result in rapid addiction by users [67,68,69]. E-cigarette use not only results in the dual use of nicotine products but also leads to nicotine and cannabis product use, a practice of growing concern to researchers as cannabis becomes legal in more countries [70,71].

Because the probability of dependence from initial nicotine use is 67%, which is the highest among drugs, including cocaine, cannabis, and alcohol, nicotine products must be controlled because of their multiple health effects [72]. Products with nicotine have rapid uptake, and addiction is sustained for a long time. Research notes that “Nicotine is the addictive compound in tobacco and is responsible for continued use of tobacco despite harms and a desire to quit…” [73]. An initial longitudinal study shows that e-cigarettes and dual use may hinder smoking cessation in real-life circumstances [28]. Though findings from neurological and respiratory system studies show immediate and long-term effects (addiction and lung damage), sometimes evidence shows increases or decreases in cardio and vascular processes contributing to specific coronary vascular diseases that are less evident on their face. Despite the lack of specific named diseases affecting heart health from e-cigarettes, an overall review of damage to vital systems often shows that risks outnumber benefits, primarily because of the adverse harms to a generation urged to use e-cigarettes as a part of a new line of nicotine-addictive products [74].

## 4. Discussion

Just as cigarette smoking is associated with many diseases, e-cigarette use has also been associated with other conditions of the skin, eyes, and teeth [74,75,76], and a higher risk of prediabetes [77]. E-cigarette use is also associated with adverse birth outcomes, although some outcome findings vary, as reported in our referenced studies. One review shows “that e-cigarette use is associated with a higher prevalence of low birth weight (LBW) (10.6%; adjusted prevalence ratio 1.88; 95% Cl 1.38–2.57) and preterm birth (12.4%; adjusted prevalence ratio 1.69; 95% Cl 1.20–2.39)” [78]. These associations may become further delineated as research shows the consequences of long-term e-cigarette use.

The alarm over e-cigarettes stems from safety concerns and the consequences of e-cigarette availability to the public, especially youth. Multiple researchers have concluded that e-cigarettes are unsafe for youth and that allowing them to be marketed as consumer products is likely to lead to increasing youth nicotine addiction and long-term consequences seen in current research evidence [79,80]. Children’s health has long been a concern in tobacco control [81]. Studies of young adult susceptibility to e-cigarette use have shown high levels of susceptibility in four diverse countries. “Susceptibility to e-cigarette use was seen among 54% of respondents from Australia, 61% from India, 62% from the UK, and 82% from China. Tobacco use, exposure to advertising, higher income, and having friends and family members who vape were associated with susceptibility to e-cigarette use”, while perceptions of harmfulness and education were negatively associated with susceptibility. “Researchers suggest that timely interventions are needed that focus on potential harms and that restricting e-cigarette advertising could reduce susceptibility levels” [82]. A study of “susceptibility to e-cigarettes in Thai students showed factors of susceptibility, including current cigarette smoking (AOR 4.28, 95% CI: 2.05–8.94), parental e-cigarette use (AOR 6.08, 95% CI: 2.81–13.17), peer e-cigarette use (AOR 3.82, 95% CI: 2.19–6.65), peer approval of smoking (AOR 1.95, 95% CI: 1.11–3.41), and unawareness of e-cigarettes’ risk (AOR 5.25, 95% CI: 2.67–10.34). The prevalence of ever e-cigarette use was 7.2%, and current e-cigarette use was 3.7% in this 2021 study of middle school students” [83]. A vital factor for children and young adults is misinformation and a lack of information. Research shows that children and young adults have a limited understanding of the nicotine in electronic cigarettes and the seriousness of nicotine addiction for their health. They do not understand that early e-cigarette use has consequences for continued nicotine addiction throughout adulthood [4].

Switching addicted users from combustible cigarettes to e-cigarettes does not eliminate addiction since nicotine addiction to e-cigarettes often remains, and both combustible and electronic cigarettes produce combined harms. Also, research does not always show that e-cigarettes are better than recommended treatment regimens [84,85].

Because e-cigarettes are harming users through biological damage and new nicotine addictions, Australia and the US have taken actions to restrict e-cigarette use. Australia is banning electronic cigarettes from entering the country [86]. The Food and Drug Administration has increasingly restricted flavored e-cigarettes in the US. It is now clamping down on unapproved disposable e-cigarettes since sales of products like Juul e-cigarettes have declined in sales after regulatory controls [87,88].

A recent review of the countries with positions on alternative tobacco products shows that 109 of 130 have chosen bans or restrictions on alternative products, including electronic cigarettes [89]. Research by Giovacchini on e-cigarette public health harms concludes: “Upon review of the currently available literature, the negative effects of e-cigarette use seem to outweigh any potential benefit… particularly given the emerging adverse effects on lung health and the potential future public health effects of e-cigarette adoption among a burgeoning new generation of tobacco product users” [90].

## 5. Limitations and Challenges in This Review

Although this review does not cover all the harms of e-cigarettes, it illustrates more advanced evidence based on systematic reviews in numerous quality studies based on research inclusion criteria. These collective reviews are performed to raise statistical certainty that the findings of the reviews are highly probable. Thus, the findings of health effects are not simply an explanation of established knowledge or a continuation of existing regulatory positions. New findings reviewed and analyzed in studies of the heart, brain, and lungs show that despite repeated assertions of safety by e-cigarette proponents, there is significant evidence of damage to the body’s vital systems from e-cigarettes. Fortunately, more evidence continues to be produced using new frameworks of investigation [36] and new tools, like Positron Emission Tomography (PET) and the Human Vaping Mimetic Real-Time Particle Analyzer (HUMITIPAA), which provide detailed findings of real-time consequences of e-cigarette use [33,37]. Researchers in public health and tobacco control communities should continue to provide evidence on e-cigarette safety.

Despite their limitations, narrative reviews can provide updates and breakthroughs that will improve investigations of safety and effectiveness. Perhaps the most significant limitation to the growing evidence about e-cigarettes is the framing of the investigation. Introducing an innovation like the e-cigarette is often used as a disruptive technology to divert attention from a core issue, such as the prevention and cessation of nicotine addiction [91]. A new technology is usually tested before the public has access to it. Neither the e-cigarette nor its constituents, which differ from those in conventional cigarettes, have been thoroughly tested and approved for use/consumption by the public. Thus, the evidence of safety for electronic cigarettes is unsystematic, while commercial e-cigarette supporters have funded and produced some evidence of beneficial results without the provision of independent studies. As with studies of combustible cigarettes and secondhand smoke, commercial nicotine interests have developed and proposed evidentiary standards to their advantage.

The tobacco industry’s role, or in this case, the nicotine product industry, is particularly problematic because of e-cigarette bias and misinformation. A recent review of the impact of a conflict of interest (COI) in e-cigarette publications reported that “any COI increased by the odds (OR 4.70; 95% CI [2.89, 7.65]) of having a positive result for vaping by 4.7 times. COI with the tobacco and pharmaceutical industries increased the odds of favorable results by 29 times (OR 29.95; 95% CI [9.84, 90.98]) and two times (OR 2.87; 95% CI [1.45, 5.69]), respectively.” Researchers concluded that a “COI should be considered and caution should be exercised when using data for policy-making” [92].

**Table 1:** Respiratory system research of electronic cigarette harms including the title, type of review, first author, reference number in the manuscript, journal and year, and a brief summary of the review’s significant findings (N = 26).

Title	Review Type	Authors/Ref No.	Journal/Year	Significance of Finding
“The Impact of E-Cigarette Exposure on Different Organ Systems: A Review of Recent Evidence and Future Perspectives.”	Narrative Review	Ali, N. et al. [8]	*J. Hazard. Mater.* **2023**	“The summarized data in this review indicate that while e-cigs use causes less adverse effects on different organs compared to traditional cigs, its long-term exposure may lead to serious health effects.”
“Impact of Vaping on Respiratory Health.”	Narrative Review	Jonas, A. [9]	*BMJ.* **2022**	This study reviews “the clinical manifestations of vaping related lung injury, including the EVALI outbreak, as well as the effects of chronic vaping on respiratory health and COVID-19 outcomes.”
“Association Between E-Cigarettes and Asthma in Adolescents: A Systematic Review and Meta-Analysis.”	Systematic Review	Li, X. et al. [10]	*Am. J. Prev. Med.* **2022**	“This study systematically evaluated the potential association between E-cigarette use and asthma in adolescents.”
“The Rise of Electronic Nicotine Delivery Systems and the Emergence of Electronic-Cigarette-Driven Disease.”	Narrative Review	McAlinden, K.D. et al. [11]	*Am. J. Physiol. Lung Cell. Mol. Physiol.* **2020**	“E-cigarette vapor is shown to affect numerous cellular processes, cellular metabolism, and cause DNA damage... E-cigarette use is associated with a higher risk of developing crippling lung conditions such as chronic obstructive pulmonary disease (COPD)….”
“Association of Electronic Cigarette Use with Respiratory Symptom Development among U.S. Young Adults.”	Narrative Review	Xie, W. et al. [12]	*Am. J. Respir. Crit. Care Med.* **2022**	“In this nationally representative cohort of young adults, former and current e-cigarette use was associated with higher odds of developing wheezing-related respiratory symptoms, after accounting for cigarette smoking and other combustible tobacco product use.”
“E-Cigarette Use: Device Market, Study Design, and Emerging Evidence of Biological Consequences”	Narrative Review	Snoderly, H.T. et al. [13]	*Int. J. Mol. Sci.* **2021**	“…current literature reports that electronic cigarette use is associated with both acute lung injury and subclinical dysfunction to the lung and vasculature that may result in pathology following chronic use. E-cigarettes can alter vascular dynamics, polarize innate immune populations towards a proinflammatory state, compromise barrier function in the pulmonary endothelium and epithelium, and promote pre-oncogenic phenomena.”
“Oral and Systemic Health Implications of Electronic Cigarette Usage as Compared to Conventional Tobacco Cigarettes: A Review of the Literature.”	Narrative Review	Abbott, A.J. et al. [15]	*J. Dent. Hyg.* **2023**	“According to current research, e-cigarettes may induce less harm than traditional tobacco products, but e-cigarettes do not remove the carcinogenic and toxic risk that has been associated with conventional cigarettes. Further research is needed to make broad conclusions on the safety of e-cigarettes compared to conventional cigarettes and to nonusers.”
“Electronic Cigarette Use, Misuse, and Harm.”	Narrative Review	Kathuria H. [16]	*Med. Clin. North Am.* **2022**	“Studies show that e-cigarette use can increase the risk to nicotine dependence and combustible tobacco use. Studies show an association between e-cigarette use and pulmonary disease. Some studies suggest reduced harm from e-cigarette use compared with smoking, but this requires further study. Most adults who use e-cigarettes also smoke cigarettes; epidemiologic studies suggest that the combination of e-cigarettes and cigarettes is more harmful than using either product alone.”
“Immediate Physiological Effects of Acute Electronic Cigarette Use in Humans: A Systematic Review and Meta-analysis.”	Systematic Review	Larue, F. et al. [17]	*Respir. Med.* **2021**	This study is a “meta-analysis of 27 studies assessing a wide range of physiological acute effects of e-cigarettes.”
“Vaping and Lung Inflammation and Injury”	Narrative Review	Park, J.A. et al. [18]	*Annu. Rev. Physiol.* **2022**	“This comprehensive review discusses the diverse spectrum of vaping exposures, epidemiological and clinical reports, and experimental findings to provide a better understanding of EVALI and the adverse health effects of chronic e-cigarette exposure.”
“E-Cigarette Use, Small Airway Fibrosis, and Constrictive Bronchiolitis.”	Narrative Review	Hariri, L.P. et al. [20]	*NEJM Evid.* **2022**	The authors “associate the histopathologic pattern of small airway–centered fibrosis, including constrictive bronchiolitis, with vaping, potentially defining a clinical and pathologic entity associated with e-cigarette use.”
“Biomarkers of Airway Immune Homeostasis Differ Significantly with Generation of E-Cigarettes.”	Narrative Review	Hickman, E. et al. [21]	*Am. J. Respir. Crit. Care Med.* **2022**	“Results indicate disrupted immune homeostasis in fourth-generation e-cigarette users and demonstrate that the biological effects of fourth-generation e-cigarette use are unique compared with those associated with previous-generation e-cigarettes.”
“Health Effects of Electronic Cigarettes: An Umbrella Review and Methodological Considerations.”	Narrative Review	Travis, N. et al. [22]	*Int. J. Environ. Res. Public Health.* **2022**	“This umbrella review underscores the urgent need for systematic reviews with better adherence to established reporting guidelines, consistent definitions of duration of e-cigarette use, a focus on newer devices, and accounting for the impacts of former or current smoking.”
“Systemic Biomarkers of Exposure Associated with ENDS Use: A Scoping Review.”	Scoping Review	Hiler, M. et al. [23]	*Tob. Control.* **2023**	Twenty-seven studies that met the study criteria showed that “Biomarkers of most volatile organic compounds are lower in electronic nicotine delivery system (ENDS) users compared with cigarette smokers…. Evidence comparing metal exposures from exclusive ENDS use, cigarette smoking and dual use is mixed and depends on the metal.”
“Pulmonary Health Effects of Electronic Cigarettes: A Scoping Review.”	Scoping Review.	Gugala, E. et al. [24]	*Health Promot. Pract.* **2022**	“Evidence indicates that EC use, especially dual use, leads to negative pulmonary effects and adverse outcomes. Education on the potential risks and publishing of EC ingredients on labels could help improve public health safety communication and reduce EC use.”
“The Role of Acrolein for E-Cigarette Vapour Condensate Mediated Activation of NADPH Oxidase in Cultured Endothelial Cells and Macrophages.”	Narrative Review	Kuntic, I. et al. [25]	*Pflugers Arch.* **2023**	The authors note that “To better understand oxidative stress mechanisms, we have exposed cultured endothelial cells and macrophages to condensed E-cigarette vapour (E-cigarette condensate) and acrolein. In both endothelial cells (EA.hy 926) and macrophages (RAW 264.7), we have observed that E-cigarette condensate incubation causes cell death.”
“The Substitution of Fifty Percent of Combustible Tobacco Smoke Exposure with Either Electronic Cigarettes or Heated Tobacco Products Did Not Attenuate Acute Lung Injury in An Animal Model.”	Narrative Review	Husari, A. et al. [26]	*Nicotine Tob. Res.* **2023**	Using an animal model, “substituting 50% of daily cigarette smoke (CS) exposure by either electronic cigarette (ECIG) or heated tobacco product (HTP) exposure did not result in significant attenuation of acute lung injury.”
“The Health Effects of Real-World Dual Use of Electronic and Conventional Cigarettes versus the Health Effects of Exclusive Smoking of Conventional Cigarettes: A Systematic Review.”	Systematic Review	Pisinger, C. et al. [27]	*Int. J. Environ. Res. Public Health.* **2022**	“Existing studies indicate that dual use is at least as, or probably even more, harmful than ESCC. Due to the predominance of cross-sectional studies and the methodological weaknesses we judged the overall certainty of the evidence as ‘low certainty’.”
“Maternal Electronic Cigarette Exposure in Relation to Offspring Development: A Comprehensive Review.”	Narrative Review	Zhang, Y. et al. [30]	*Am. J. Obstet. Gynecol. MFM.* **2022**	“Research suggests that maternal e-cigarette exposure may result in compromised neurodevelopment in newborns. In summary, current evidence is insufficient to rigorously evaluate the health impacts of maternal e-cigarette use on offspring development. Future investigations are warranted.”
“Physical and mental health outcomes associated with adolescent E-cigarette use.”	Narrative Review	Livingston, J.A. et al. [31]	*J. Pediatr. Nurs.* **2022**	“Although somewhat less harmful than conventional cigarettes, e-cigarette use is related to a variety of negative physical and mental health outcomes among adolescent users.”
“Impact of Electronic Cigarettes on the Upper Aerodigestive Tract: A Comprehensive Review for Otolaryngology Providers.”	Narrative Review	Soo, J. et al. [32]	*OTO Open.* **2023**	“Although e-cigs are likely less harmful than conventional cigarettes, preliminary research on e-cigs suggest several deleterious effects including in the upper aerodigestive tract. Due to this, there has been increased interest in restricting e-cig usage, particularly among the adolescent population, and caution in recommending e-cigs to current smokers.”
“The Nicotine and Tobacco Epidemic among Adolescents: New Products are Addicting Our Youth.”	Narrative Review	Heinly, A.; Walley, S. [33]	*Curr. Opin. Pediatr.* **2023**	“Millions of adolescents continue to use nicotine and tobacco products, which puts them at risk for health problems, including nicotine addiction. Pediatric providers can provide prevention messages, screen youth for tobacco and nicotine use, and offer appropriate treatment options. Regulation of tobacco and nicotine products by the FDA is critical to reverse this public health epidemic of youth nicotine and tobacco use.”
“Molecular Imaging of Pulmonary Inflammation in Electronic and Combustible Cigarette Users: A Pilot Study.”	Narrative Review	Wetherill, R. et al. [34]	*J. Nucl. Med.* **2023**	This is the first PET imaging study to compare lung inflammation between EC and cigarette users in vivo. We found preliminary evidence that EC users have greater pulmonary inflammation than cigarette smokers and controls, with a positive association between pulmonary and peripheral measures of inflammation.
“A Review of Toxicity Mechanism Studies of Electronic Cigarettes on Respiratory System.”	Narrative Review	Wang, L. et al. [35]	*Int. J. Mol. Sci.* **2022**	This review summarizes the toxicity mechanisms and signal pathways of conventional cigarettes and e-cigarettes concerning the respiratory system, which could give researchers a better understanding and direction on the effects of e-cigarettes on our health.
“Electronic Cigarette Menthol Flavoring Is Associated with Increased Inhaled Micro and Sub-Micron Particles and Worse Lung Function in Combustion Cigarette Smokers.”	Narrative Review	Chandra, D. et al. [37]	*Respir. Res.* **2023**	The authors “demonstrate utility of the HUMITIPAA as a predictive enabling technology to identify inhalation toxicological potential of emerging ECs as the chemical formulation of e-liquid gets modified.”

**Table 2:** Cardiovascular system research of electronic cigarette harms including the title, type of review, first author, reference number in the manuscript, journal and year, and a brief summary of the review’s significant findings (N = 22).

Title	Review Type	Authors	Journal/Year	Significance of Finding
“E-Cigarettes and Cardiopulmonary Health.”	Narrative Review	Tarran, R. et al. [14]	*Function.* **2021**	“This report is prepared for clinicians, researchers, and other health care providers to provide the current state of knowledge on how e-cigarette use might affect cardiopulmonary health, along with research gaps to be addressed in future studies.”
“A Novel Role for Vaping in Mitochondrial Gene Dysregulation and Inflammation Fundamental to Disease Development.”	Narrative Review	Tommasi, S. et al. [19]	*Sci. Rep.* **2021**	The authors’ “findings accord with the growing evidence on the central role of mitochondria as signaling organelles involved in immunity and inflammatory response, which are fundamental to disease development.”
“The Cardiovascular Effects of Electronic Cigarettes.”	Narrative Review	Khadka, S. et al. [38]	*Curr. Cardiol. Rep.* **2021**	“Despite a gap in clinical studies and randomized trials analyzing adverse cardiovascular effects of e-cigarette use, the existing literature supports that different constituents of e-cigarettes such as nicotine, carbonyls, and particulate matters carry potential risk for cardiovascular diseases (CVD) on its users.”
“E-Cigarettes and Cardiopulmonary Health: Review for Clinicians.”	Narrative Review	Neczypor, E.W. et al. [39]	*Circulation.* **2022**	“Consistent with the Centers for Disease Control and Prevention recommendations, clinicians should monitor the health risks of e-cigarette use, discourage nonsmokers and adolescents from using e-cigarettes, and discourage smokers from engaging in dual use without cigarette reduction or cessation.”
“Comparable Impairment of Vascular Endothelial Function by a Wide Range of Electronic Nicotine Delivery Devices.”	Narrative Review	Rao, P. et al. [40]	*Nicotine Tob. Res.* **2022**	“A wide range of ENDS, including multiple types of e-cigarettes with and without nicotine, a heated tobacco product, and an ultrasonic vaping device devoid of heating coil, all impair FMD after a single vaping session comparably to combusted cigarettes.”
“Electronic Cigarette Use and the Risk of Cardiovascular Diseases.”	Narrative Review	Espinoza-Derout, J. et al. [41]	*Front. Cardiovasc. Med.* **2022**	“The increased hyperlipidemia, sympathetic dominance, endothelial dysfunction, DNA damage, and macrophage activation are prominent effects of e-cigarettes. Additionally, oxidative stress and inflammation are unifying mechanisms at many levels of the cardiovascular impairment induced by e-cigarette exposure. This review outlines the contribution of e-cigarettes in the development of cardiovascular diseases and their molecular underpinnings.”
“Impact of Electronic Cigarette Vaping on Cerebral Ischemia: What We Know So Far.”	Narrative Review	Siegel, J. et al. [42]	*Transl. Stroke Res.* **2022**	“The current review strives to extrapolate the existing understanding of the nicotine-induced effects of conventional smoking on the brain to the possible effects that ECs may have on the brain, which may ultimately have a potential for adverse stroke risk or severity.”
“Cardiopulmonary Consequences of Vaping in Adolescents: A Scientific Statement from the American Heart Association.”	Narrative Review	Wold, L.E. et al. [44]	*Circ. Res.* **2022**	“The goals of this scientific statement are to provide salient background information on the cardiopulmonary consequences of e-cigarette use (vaping) in adolescents, to guide therapeutic and preventive strategies and future research directions, and to inform public policymakers on the risks, both short and long term, of vaping.”
“Effects of Mango and Mint Pod-Based E-Cigarette Aerosol Inhalation on Inflammatory States of The Brain, Lung, Heart, and Colon in Mice.”	Narrative Review	Moshensky, A. et al. [45]	*Elife.* **2022**	The authors’ “findings suggest that daily e-cigarette use may cause neuroinflammation, which may contribute to behavioral changes and mood disorders. In addition, e-cigarette use may cause gut inflammation, which has been tied to poor systemic health, and cardiac inflammation, which leads to cardiovascular disease.”
“Acute Effects of Electronic Cigarettes on Vascular Endothelial Function: A Systematic Review and Meta-analysis of Randomized Controlled Trials.”	Systematic Review	Meng, X.C. et al. [46]	*Eur. J. Prev. Cardiol.* **2022**	“Evidence from authors’ pooled analyses indicated that acute inhalation of e-cigarettes leads to negative changes in vascular endothelial function. E-cigarettes cannot be used as an alternative to public health strategies for tobacco control and should not be considered cardiovascular safety products. More future research should be conducted to verify our findings.”
“Increased Vulnerability to Atrial and Ventricular Arrhythmias Caused by Different Types of Inhaled Tobacco or Marijuana Products.”	Narrative Review	Qiu, H. et al. [47]	*Heart Rhythm* **2023**	“These pathophysiological results indicate that tobacco and marijuana products can induce arrhythmogenic substrates involved in cardiac electrical, structural, and neural remodeling, facilitating the development of arrhythmias.”
“E-Cigarettes and Their Lone Constituents Induce Cardiac Arrhythmia and Conduction Defects in Mice.”	Narrative Review	Carll, A.P. et al. [48]	*Nat. Commun.* **2022**	“This study indicates that chemical constituents of e-cigarettes could contribute to cardiac risk by provoking pro-arrhythmic changes and stimulating autonomic reflexes.”
“Chronic E-Cigarette Use Impairs Endothelial Function on the Physiological and Cellular Levels.”	Narrative Review	Mohammadi, L. et al. [49]	*Arterioscler. Thromb. Vasc. Biol.* **2022**	“Chronic vaping and smoking both impair FMD and cause changes in the blood that inhibit endothelial NO release. Vaping, but not smoking, causes changes in the blood that increase microvascular endothelial permeability and may have a vaping-specific effect on intracellular oxidative state. The results suggest a role for RAGE in e-cigarette-induced changes in endothelial function.”
“Impairment of Endothelial Function by Cigarette Smoke Is Not Caused by a Specific Smoke Constituent, but by Vagal Input From the Airway.”	Narrative Review	Nabavizadeh, P. et al. [50]	*Arterioscler. Thromb. Vasc. Biol.* **2022**	“There is no single constituent or class of constituents responsible for acute impairment of endothelial function by smoke; rather, authors propose that acute endothelial dysfunction by disparate inhaled products is caused by vagus nerve signaling initiated by airway irritation.”
“Association of Volatile Organic Compound Levels With Pod-Based Electronic Cigarette-Induced Changes in Vascular Function of Young Adults.”	Narrative Review	Majid, S.A. et al. [51]	*Circulation.* **2022**	The authors’ findings demonstrated “that pod-based e-cigarette use had acute and chronic vascular effects in healthy young adults including those who never used combustible cigarettes. Select VOC metabolites were associated with the vascular changes and altered nitric oxide production suggesting relevance to vascular health.”
“Immunological Insights into Cigarette Smoking-Induced Cardiovascular Disease Risk.”	Narrative Review	Dahdah, A. et al. [52]	*Cells.* **2022**	The authors “highlight some of the important pathological mechanisms that involve cigarette smoking and its many components on cardiovascular disease and the immune systems in order to have a better understanding of the mechanisms at play.”
“Short-term Effects of Electronic Cigarettes on Cerebrovascular Function: A Time Course Study.”	Narrative Review	Mills, A. et al. [53]	*Exp. Physiol.* **2022**	“The finding that Ecig (without nicotine) and cigarette (with nicotine) exposure produce the same effects suggesting that nicotine is not likely to be triggering MCA dysfunction, and that vaping (with/without nicotine) has potential to produce the same vascular harm and/or disease as smoking.”
“Electronic Nicotine Delivery Systems and Cardiovascular/Cardiometabolic Health.”	Narrative Review	Mears, M.J. et al. [54]	*Circ. Res.* **2023**	“This review provides an overview of the cardiovascular, cardiometabolic, and vascular implications of the use of e-cigs, and the potential short- and long-term health effects. A robust understanding of these effects is important in order to inform policy makers on the dangers of e-cigs use.”
“Characterization and Summarization of the Impact of Electronic Cigarettes on the Cardiovascular System: A Systematic Review and Meta-Analysis.”	Systematic Review	Rahman, A. et al. [55]	*Cureus.* **2023**	The authors “conclude that using e-cigarettes has a detrimental effect on cardiac health. The risk of severe cardiac conditions increases with e-cigarettes. Thus, vaping can do more harm than good. Consequently, the misleading notion that e-cigarettes are less harmful should be challenged.”
“Association of Electronic Cigarette Exposure on Cardiovascular Health: A Systematic Review and Meta-Analysis.”	Systematic Review	Siddiqi, T.J. et al. [56]	*Curr. Probl. Cardiol.* **2023**	“Results demonstrate that smoking EC is associated with a significant increase in cardiovascular hemodynamic measures and biomarkers. Our findings can aid policymakers in making informed decisions regarding the regulation of EC to ensure public safety.”
“The Effect of Emerging Tobacco Related Products and Their Toxic Constituents on Thrombosis.”	Narrative Review	Alarabi, A.B. et al. [57]	*Life Sci.* **2022**	In this review, the authors “discuss the potential impact of ETRPs on thrombosis-based CVD. Specifically, we will review how these products and the major chemicals they produce and/or emit can trigger key players in the process of thrombosis, namely inflammation, oxidative stress, platelets, coagulation, and the vascular endothelium, and the relationship between these effects.”
“E-Cigarette Toxicology.”	Narrative Review	Gordon, T. et al. [58]	*Annu. Rev. Pharmacol. Toxicol.* **2022**	“Adverse health effects related to e-cigarette aerosols are influenced by several factors, including e-liquid components, physical device factors, chemical changes related to heating, and health of the e-cigarette user (e.g., asthmatic). Federal, state, and local regulations have attempted to govern e-cigarette flavors, manufacturing, distribution, and availability, particularly to underaged youths.”

**Table 3:** Neurological system research of electronic cigarette harms including the title, type of review, first author, reference number in the manuscript, journal and year, and a brief summary of the review’s significant findings (N = 16).

Title	Review Type	Authors	Journal/Year	Significance of Finding
“Longitudinal Transition Outcomes Among Adult Dual Users of E-Cigarettes and Cigarettes with The Intention to Quit in The United States: PATH Study (2013–2018).”	Narrative Review	Osibogun, O. et al. [28]	*Prev. Med. Rep.* **2022**	“Findings show that in a real-world scenario, dual e-cigarette and cigarette use may hinder rather than facilitate smoking cessation among those interested in quitting. This needs consideration when assessing the population impact of e-cigarettes and their role in harm reduction.”
“E-Cigarettes, Nicotine, The Lung and The Brain: Multi-Level Cascading Pathophysiology.”	Narrative Review	Herman, M. et al. [59]	*J. Physiol.* **2020**	The authors conclude “that vaping needs to be studied by multi-disciplinary teams that include pulmonary and neurophysiologists as well as behaviourists and addiction specialists to fully understand their impact on human physiology.”
“Nicotine Gateway Effects on Adolescent Substance Use.”	Narrative Review	Ren, M. et al. [60]	*West J. Emerg. Med.* **2019**	The authors present “a large collection of clinical and preclinical evidence that adolescent nicotine exposure influences long-term molecular, biochemical, and functional changes in the brain that encourage subsequent drug abuse.”
“Evidence of Altered Brain Responses to Nicotine in an Animal Model of Attention Deficit/Hyperactivity Disorder.”	Narrative Review	Poirier, G.L. et al. [61]	*Nicotine Tob. Res.* **2017**	“In the SHR, nicotine triggered an atypical response in one VTA circuit while normalizing activity in another. The VTA has been widely implicated in drug reward. Data suggest that increased susceptibility to nicotine addiction in individuals with ADHD may involve altered responses to nicotine involving VTA circuits.”
“Substance Use and Nicotine Dependence in Persistent, Remittent, and Late-Onset ADHD: A 10-year Longitudinal Study from Childhood to Young Adulthood.”	Narrative Review	Ilbegi, S. et al. [62]	*J. Neurodev. Disord.* **2018**	“SUD and nicotine dependence are associated with a negative ADHD outcome. Results further emphasize the need for clinicians to comprehensively assess substance use when diagnosing ADHD in adolescents and adults.”
“Brain Lesions Disrupting Addiction Map to A Common Human Brain Circuit.”	Narrative Review	Joutsa, J. et al. [63]	*Nat. Med.* **2022**	The authors conclude “that brain lesions disrupting addiction map to a specific human brain circuit and that hubs in this circuit provide testable targets for therapeutic neuromodulation.”
“Rapid Brain Nicotine Uptake from Electronic Cigarettes.”	Narrative Review	Solingapuram Sai, K.K. et al. [64]	*J. Nucl. Med.* **2020**	“E-cigs can deliver nicotine to the brain with a rapidity similar to that of C-cigs. Therefore, to the extent that rapid brain uptake promotes smoking reward, E-cigs might maintain a degree of nicotine dependence and also serve as a noncombustible substitute for cigarettes.”
“Longitudinal Assessments of Neurocognitive Performance and Brain Structure Associated with Initiation of Tobacco Use in Children, 2016 to 2021.”	Narrative Review	Dai, H.D. et al. [65]	*JAMA Netw. Open.* **2022**	“In this cohort study, initiating tobacco use in late childhood was associated with inferior cognitive performance and reduced brain structure with sustained effects at 2-year follow-up. These findings suggest that youths vulnerable to e-cigarettes and tobacco products should be treated as a priority population in tobacco prevention.”
“Prospectively Assessed Long-Term Outcomes of Patients with E-Cigarette- or Vaping-Associated Lung Injury.”	Narrative Review	Blagev, D.P. et al. [66]	*Ann. Am. Thorac. Soc.* **2022**	“Patients with EVALI, despite their youth, commonly have significant long-term respiratory disability; cognitive impairment; symptoms of depression, anxiety, post-traumatic stress; and persistent vaping.”
“Exploring the Gateway Hypothesis of E-cigarettes and Tobacco: A Prospective Replication Study among Adolescents in the Netherlands and Flanders.”	Narrative Review	Martinelli, T. et al. [67]	*Tob. Control.* **2023**	“This study replicated the positive relation between e-cigarette use and tobacco smoking in both directions for adolescents. This may mean that the gateway works in two directions, that e-cigarette and tobacco use share common risk factors, or that both mechanisms apply.”
“Electronic Nicotine Delivery Systems Use Predicts Transitions in Cigarette Smoking among Young Adults.”	Narrative Review	Loukas, A. et al. [68]	*Drug Alcohol Depend.* **2022**	“ENDS use in young adulthood increases the risk for cigarette smoking behaviors across the continuum of uptake and progression. Prevention and cessation efforts targeting both ENDS and cigarette use during young adulthood are needed.”
“Effects of Vaping on Uptake and Cessation of smoking: Longitudinal Analysis in Aotearoa New Zealand Adults.”	Narrative Review	Mason, A. et al. [69]	*Drug Alcohol Rev.* **2023**	“The findings demonstrate that vaping appeared to be just as likely to have a gateway effect to smoking as it was to have a cessation effect. This highlights the need for greater consideration regarding vaping-related policies and restrictions.”
“Cannabis Vaping Among Youth and Young Adults: A Scoping Review.”	Scoping Review	Harrell, M.B. et al. [70]	*Curr. Addict. Rep.* **2022**	“Cannabis vaping is increasingly common among youth and young adults and more prevalent is settings where recreational use for adults has been legalized. The literature documents a number of negative health effects of cannabis vaping for young people, along with risk factors and reasons for the same.”
“Dual Use of Nicotine and Cannabis Through Vaping Among Adolescents.”	Narrative Review	Moustafa, A.F. et al. [71]	*Am. J. Prev. Med.* **2022**	“From middle to late adolescence, vaping of nicotine and cannabis develop in close parallel. Regulatory policy and prevention interventions should consider the interplay between these 2 substances during this period of adolescence.”
“Probability and Predictors of Transition from First Use to Dependence on Nicotine, Alcohol, Cannabis, and Cocaine: Results of The National Epidemiologic Survey on Alcohol and Related Conditions (NESARC).”	Narrative Review	Lopez-Quintero, C. et al. [72]	*Drug Alcohol Depend.* **2011**	“The existence of common predictors of transition dependence across substances suggests that shared mechanisms are involved. The increased risk of transition to dependence among individuals from minorities or those with psychiatric or dependence comorbidity highlights the importance of promoting outreach and treatment of these populations.”
“Tobacco and Nicotine Use.”	Narrative Review	Le Foll, B. et al. [73]	*Nat. Rev. Dis. Primers.* **2022**	“Efforts associated with innovative policy regulations (aimed at reducing nicotine content or eliminating tobacco products) have the potential to reduce the prevalence of tobacco and nicotine use and their enormous adverse impact on population health.”

## 6. Conclusions

This review has presented recent research of significance in addressing the epidemic of e-cigarette use that has developed from the aggressive efforts of tobacco industry interests to further the use of electronic cigarettes. These investigations must continue since existing findings are frequently tentative due to the changing nature of the types, e-liquids, and status of e-cigarettes worldwide.

This paper highlights the following:

- Recent evidence shows that e-cigarettes are unsafe because of research findings by those studying the effects of e-cigarette use on the respiratory, cardiovascular, and neurological systems. Nicotine addiction is the central driving force of the adverse health effects of new alternative nicotine products. The addictive nature of the drug nicotine necessitates its restriction to protect public health.
- The rejection of e-cigarette products as consumer goods is essential for preventing e-cigarette nicotine addiction on a population level.

The effects on bodily systems provide information necessary for building or refining approaches and policies on electronic cigarette use. Medical evidence shows that e-cigarettes cause harm to systems of the body. Consequently, it makes sense to ban or tightly regulate e-cigarettes. Unfortunately, those who have a financial interest in e-cigarette sales prefer to represent e-cigarette use as safe and effective regardless of the accumulating evidence from health researchers and medical practitioners and likely health effects.

Based on emerging evidence, we believe that an approach to protect health should not view harm as a matter of balancing competing interests but facing the cumulative scientific evidence and acting to prevent exposures that cause disease and death. Tobacco control is best served by instituting health promotion policies that work to eliminate alternative nicotine products. Health protection and promotion must move beyond perpetuating the highly addictive drug nicotine.
